# The Aphasia Recovery Cohort, an open-source chronic stroke repository

**DOI:** 10.1038/s41597-024-03819-7

**Published:** 2024-09-09

**Authors:** Makayla Gibson, Roger Newman-Norlund, Leonardo Bonilha, Julius Fridriksson, Gregory Hickok, Argye E. Hillis, Dirk-Bart den Ouden, Christopher Rorden

**Affiliations:** 1 Department of Psychology, University of South Carolina, Columbia, SC USA; 2 Department of Neurology, University of South Carolina School of Medicine, Columbia, SC USA; 3 Department of Communication Sciences and Disorders, University of South Carolina, Columbia, SC USA; 4 Department of Cognitive Sciences, University of California, Irvine, CA USA; 5 Department of Neurology, John Hopkins University, Baltimore, MD USA

**Keywords:** Databases, Research data, Stroke

## Abstract

Sharing neuroimaging datasets enables reproducibility, education, tool development, and new discoveries. Neuroimaging from many studies are publicly available, providing a glimpse into progressive disorders and human development. In contrast, few stroke studies are shared, and these datasets lack longitudinal sampling of functional imaging, diffusion imaging, as well as the behavioral and demographic data that encourage novel applications. This is surprising, as stroke is a leading cause of disability, and acquiring brain imaging is considered standard of care. The first release of the Aphasia Recovery Cohort includes imaging data, demographics and behavioral measures from 230 chronic stroke survivors who experienced aphasia. We also share scripts to illustrate how the imaging data can predict impairment. In conclusion, recent advances in machine learning thrive on large, diverse datasets. Clinical data sharing can contribute to improvements in automated detection of brain injury, identification of white matter hyperintensities, measures of brain health, and prognostic abilities to guide care.

## Background & Summary

Sharing neuroimaging data enables reproducibility and reuse, and modern machine learning methods can aggregate across diverse datasets to disentangle correlated factors^1,2^. Thousands of neuroimaging datasets are now available spanning progressive disorders, healthy young adults, brain development, and aging^3–6^. However, there are few open datasets for stroke, despite the fact that stroke is a leading cause of disability^7^ and brain imaging at admission is standard of care^8^. Furthermore, neuroimaging has proven a powerful tool for understanding the consequences of stroke^9–13^. Here we describe the Aphasia Recovery Cohort (ARC), which already provides longitudinal neuroimaging, demographic, and behavioral data for 230 unique individuals with stroke observed across a total of 902 scanning sessions. We describe existing and potential studies that can leverage shared stroke datasets. Furthermore, we provide a basic guide for the considerations and challenges involved in creating shared neuroimaging datasets of clinical populations.

### The potential of open data

The emphasis, infrastructure, and formats for sharing and analyzing neuroimaging datasets have matured rapidly. For example, the Image Data Archive^3^ stores neuroscience data from 151 studies and 96,558 subjects, including progressive disorders like Alzheimer’s disease^4^ and frontotemporal dementia^5^, as well as data from healthy adults captured from the Human Connectome Project^6^. Likewise, OpenNeuro^14^ shares 922 datasets from 35,946 participants using the Brain Imaging Data Structure (BIDS) format^15^ to define imaging modalities, participants, and sequence details. This combination of open and structured data enables Findable, Accessible, Interoperable, and Reusable (FAIR) principles to reduce the accessibility barrier^16^. Open data allows aggregated analyses that are able to model correlated variables. New tools like BrainLife^17^, COINSTAC^18^, and Neurodesk^19^ are able to scale on demand to process large datasets with precise version control to allow reproducible and collaborative neuroscience. These methods reduce the conventional barriers including data access and compute resources for large-scale analyses, allowing scientists at under-resourced institutions to make novel discoveries. BrainLife, for example, only requires access to an internet-connected computer with a web browser to import data directly from OpenNeuro, preprocess the data, and compute results using existing cloud infrastructure, providing efficiency and transparency for neuroimaging research^17^.

### The challenges of open data

While sharing data has clear benefits for the neuroimaging community, it also poses a burden for the teams that acquire data. Curating and anonymizing data is arduous. Generalizing demographics and defacing images to remove recognizable features must be done judiciously to ensure thorough removal of Protected Health Information (PHI) without biasing subsequent analyses. Indeed, imaging teams may find sharing data counterproductive, as they have spent considerable resources acquiring it, and sharing may only benefit competitors who can simply focus on leveraging existing data. On the other hand, major public funding agencies often encourage data sharing. For example, in 2023, the National Institute of Health (NIH) introduced data management and sharing plans, which allow a budget for data curation and sharing. Therefore, the present work provides a timely description of the challenges and solutions for sharing clinical neuroimaging data.

### Challenges of open stroke imaging data

There are few existing shared neuroimaging datasets of stroke. The seminal Anatomical Tracings of Lesions After Stroke (ATLAS)^20^, which has grown to include T1-weighted MRI scans and manually segmented lesion drawings from 1271 individuals from 44 sites located in 11 countries, includes data from multiple scanner manufacturers (GE, Philips, and Siemens), multiple field strengths (1.5 and 3 T) and variable days post stroke (1..10806). However, since the goal of this dataset is to improve automated lesion segmentation algorithms, no other demographic or behavioral data are shared, limiting opportunities for discovery (though note that the parent ENIGMA Stroke Recovery Working Group does attempt to harmonize some impairment measures, and external teams can access these if the team acquiring the data agrees^21^). Likewise, the shared CQ500 dataset includes 491 CT scans with radiological findings including stroke and provides a gold standard for training machine learning algorithms, but does not include impairment or demographic measures^22^. Similarly, several recent data challenges^23,24^ have provided structural scans of stroke patients where a subset includes lesion maps, again sufficient for improving lesion mapping methods. Faria and colleagues^25^ provide hospital admission MRI scans from 2888 acute stroke patients that include demographic information, basic clinical profile (NIH Stroke Scale/Score), hospitalization duration, blood pressure at admission, body mass index, and associated health conditions. This dataset includes diffusion scans that can be used to train algorithms to detect acute lesions^26^, and the behavioral data can be used to explore stroke related impairments. This rich dataset provides a large, diverse sample of acute stroke data. However, to address privacy concerns, it is shared under strict guidelines and restrictions, which require institutional review and demand that any computers on which the data are housed be disconnected from the internet. This necessarily prevents the use of scalable resources such as BrainLife and makes this dataset suboptimal for educational purposes. Beyond these explicitly shared datasets, stroke neuroimaging datasets often see re-use via direct sharing between teams. For example, Xiong *et al*.^27^ explored verbal fluency in an archival analysis of the Cognition And Neocortical Volume After Stroke (CANVAS) study^28^. Scientists can purchase access to the UK Biobank which provides access to scans from thousands of healthy individuals, some of whom had strokes after recruitment^29^, providing a unique opportunity to determine predictors of brain injury. The Predict Language Outcome and Recovery After Stroke (PLORAS) includes structural and functional imaging data as well as a wide range of standardized scores from stroke survivors, however access is limited to relevant members of the PLORAS Research Team and local collaborators^30^.

A limitation of most previously shared datasets of stroke is that they only provide anatomical scans, and not modalities that can be used to infer brain function (e.g., functional and resting-state MRI) or directly map fiber bundle tractography (though one can infer disconnections from anatomical scans^31^). While anatomical scans reveal regions that are damaged, they may fail to detect regions that are disconnected and non-functional. Another unique feature of our dataset relative to previously shared clinical datasets is the inclusion of multiple sessions across individuals, with the same individuals seen across months and years. This longitudinal aspect allows scientists to model lesion expansion^32^ and its consequences as well as providing insight into the compensation mechanisms that are observed in the chronic stages of stroke recovery.

In short, while there are hundreds of open neuroimaging datasets for healthy adults, there are relatively few for stroke. Basic neuroimaging often restricts the age of participants and the methods to minimize variability. In contrast, clinical studies should reflect the heterogeneity of the population (e.g., age at time of stroke can predict impairment^33^). Therefore clinical neuroimaging may particularly benefit from the statistical power provided by aggregate analysis across datasets. Sample diversity is also crucial to ensuring that predictive tools are not overtrained on people of European ancestry^34^. With regard to stroke, risk factors like obesity, diabetes, and small vessel disease impact incidence and outcome. Furthermore, image alignment and positioning in the head coil is often impacted by kyphosis, leading to poor starting estimates for many automated image preprocessing routines. Clinical scans often include features such as lesions, wide diploic space, and variations in skull shape that can disrupt automated image processing tasks such as spatial normalization and segmentation. While our team develops and shares wrappers for Statistical Parametric Mapping (SPM) that are robust to these features^35^, we have intentionally decided to share both the raw and processed data. Our rationale is that this allows others to devise robust tools for these features.

Creating open datasets requires protecting participant privacy and respecting regional privacy regulations. For example, the European Union (EU) General Data Protection Regulation (GDPR) is, in general, more stringent than the Health Insurance Portability and Accountability Act (HIPAA) of the United States (US)^36^. The International Safe Harbor Privacy Principles attempt to ease these issues and provide guidelines for generalizing demographic variables that can identify individuals^37^. These processes of generalizing and removing personally identifying features can protect privacy but can also influence the statistical power of a research study^38,39^. Therefore, there is a tension between robustly de-identifying data and maximizing the potential of a dataset. For example, public datasets typically remove facial features from anatomical MRI scans to protect privacy, but this step can impair subsequent processing, including segmentation^39^, brain age estimations^40^, and unified normalization-segmentation^41^. On the other hand, simple defacing algorithms can preserve enough information to allow some level of facial recognition^41^. Furthermore, as re-identification methods become more sophisticated, it is hard to predict if any particular method of data generalization provides future-proof anonymization^1^. A related issue is the distinction between retrospective archival observational studies of medical records where consent is not sought and prospective studies like the Aphasia Recovery Cohort, where participants actively provide informed consent but which can incur a selection bias^42^. While some data acquired without direct consent can be adequately anonymized such that they no longer qualify as human subjects data, institutional or Institutional Review Board (IRB) policies may still prohibit sharing for secondary purposes^43^. In general, many in the public express concerns surrounding trust, transparency, and privacy with regard to sharing health data.

These challenges demonstrate that there cannot be a one-size-fits-all solution for sharing clinical datasets. Many teams will be unable to share clinical data due to local regulations, enrollment, and consent criteria, as well as costs associated with carefully curating and anonymizing data. However, in the long term, recognizing these challenges can help many teams develop strategies compatible with data sharing of upcoming prospective studies. While these obstacles may ultimately limit the number of shared clinical repositories, there is a clear need for open stroke repositories that allow tool developers to ensure their algorithms are robust when faced with diverse clinical datasets. Likewise, at least a few truly open datasets are crucial for education, allowing tutorials to train new users with real world data. Here we introduce the Aphasia Recovery Cohort, published to meet this need.

### The aphasia recovery cohort

Although stroke has numerous consequences, the current neuroimaging dataset is limited to individuals with chronic aphasia. Aphasia is seen in roughly one-third of the over 25 million individuals globally who experience a stroke each year, with many of these individuals still experiencing symptoms a year later^44,45^. The Aphasia Recovery Cohort (ARC) repository is designed to fill the niche of a truly open resource for reproducibility, re-use, tool refinement, and education. We developed this dataset by combining data from a number of previous studies. These include anomia treatment^46^, POLAR (Predicting Outcome of Language Rehabilitation in Aphasia) protocol^47^, speech entrainment^47,48^, and a randomized clinical trial to examine the influence of brain stimulation on aphasia^49^.

## Methods

### Cohort

Participants included in the Aphasia Recovery Cohort were recruited for participation in various studies, and provided informed consent approved by the Institutional Review Board, located in Columbia, SC, at the University of South Carolina (USC). Enrollment requirements included individuals who had experienced a left-hemisphere stroke at least 6 months (or 12 months for some studies) prior to enrollment, between the ages of 21 and 80 years old, with no contraindications to MRI or additional neurological impairments (such as multiple sclerosis, Parkinson’s, dementia, etc). By default, and due to the evolution of studies within the Aphasia Lab at USC, this repository quickly became longitudinal. While some studies were designed as longitudinal clinical trials, many participants participated in multiple studies. The initial tranche of the dataset includes a total of 230 participants, comprising 89 females and 141 males. Demographic distribution included 2 participants identified as Asian, 47 identified as Black/African American, and 181 as White. Aphasia types were categorized as follows: 62 with Anomic aphasia, 85 with Broca’s aphasia, 26 with Conduction aphasia, 14 with Global aphasia, 31 with no diagnosed aphasia, 4 with Transcortical Motor aphasia, and 7 with Wernicke’s aphasia. The average age of stroke onset was 58 years old, with a range from 27 to 80 years. Days post-stroke from the date of the Western Aphasia Battery (WAB) assessment ranged from a minimum of 183 days to a maximum of 7998 days, with an average of 1414 days post-stroke. The diversity in time post-stroke within the Aphasia Recovery Cohort represents the dataset’s composition, which includes individuals at different stages of stroke recovery. This variability in post-stroke duration is characteristic of longitudinal studies, concentrating on chronic stroke recovery and mirrors the natural evolution of the condition over time.

The IRB at the University of South Carolina determined that the anonymized human data, with variables generalized to meet safe harbor criteria, was exempt from further review (proposal ‘OpenNeuro Clinical’ Pro00132576).

### Design: behavioral measures

The most commonly used and well-known assessment of aphasia in the US, the Western Aphasia Battery (WAB) and Western Aphasia Battery-Revised (WAB-R)^50^, is included in this repository. For a subset of the participants, the scores are from the original assessment (WAB)^51^, whereas the majority of participants were tested with the Western Aphasia Battery-Revised (WAB-R)^52^. This assessment consists of several sub-scores identifying distinct language impairment characteristics seen across aphasia types. Note that the WAB and WAB-R are essentially the same test, with only very minor variations and having a weighted score that emphasizes spoken language difficulties over comprehension. We share the categorical Aphasia types that were established in accordance with battery procedures and include the following: anomic, Broca’s, conduction, global, transcortical motor, transcortical sensory, Wernicke’s, and not aphasic. The provided sub-scores assess distinct aspects of aphasia, encompassing Fluency (range: 0–10) and Auditory Comprehension (range: 0–10). Additionally, we compute a summary score reflecting the overall severity of aphasia, termed Aphasia Quotient (WAB-AQ; range: 0–100).

### MRIs

MRI data for this study were obtained using Siemens 3 T MRI scanners. As the core experiments spanned several years, adjustments to sequence parameters were adapted over time. Likewise, the original Trio scanners with 12-channel head coils were upgraded to the Prisma configuration using the 20-channel head/neck coil. As all images are shared in BIDS format sequence parameters such as these are provided for each image.

The initial visit of each individual typically includes one T1-weighted 3D MP-RAGE and one T2-weighted SPACE image as described by Yourganov *et al*.^53^, this pair of series used 1 mm isotropic with identical volume centers and slice angulation. Several diffusion-imaging sequences were acquired, including those suitable for microstructural measures as described by McKinnon *et al*.^54^ and those suitable for connectivity measures^55^. Resting-state images can be used to infer functional connectivity, as described by Yourganov *et al*.^56^. A sparse-fMRI sequence is used to acquire functional activation data, where participants attempt to name pictures of objects while remaining silent when shown abstract art, as described by Fridriksson^46^.

### Post-processed images

Beyond the raw data, we also provide post-processed images. Specifically, to demonstrate the potential of the aggregated data and provide a simple introduction for education, we provide minimal scripts and processed images (https://github.com/neurolabusc/AphasiaRecoveryCohortDemo). These images are aligned to standard MNI152 space using the clinical toolbox for SPM^35^.

**Fig. 1:**
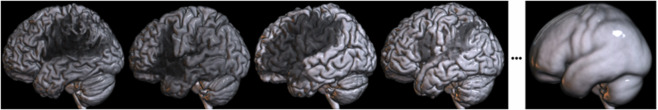
Our scripts spatially normalize and brain-extract the anatomical scans for each participant. The scripts generate a volume rendering for each individual’s image (left). Since the unified segmentation-and-normalization process creates a virtuous cycle, better alignment results in tissue identification. This surface rendering provides a simple method to ensure that the shape and size of each individual’s brain have been accurately aligned. The scripts also create mean images (right) for the entire population.

Here we describe these scripts. These scripts are able to identify all of the lesion maps and the anatomical images they were drawn on. The anatomical images and lesion maps are then spatially normalized to a common template image using our Clinical Toolbox for SPM^35^. This toolbox prevents the presence of the injury from disrupting this coregistration process (Fig. 1).

**Fig. 2:**
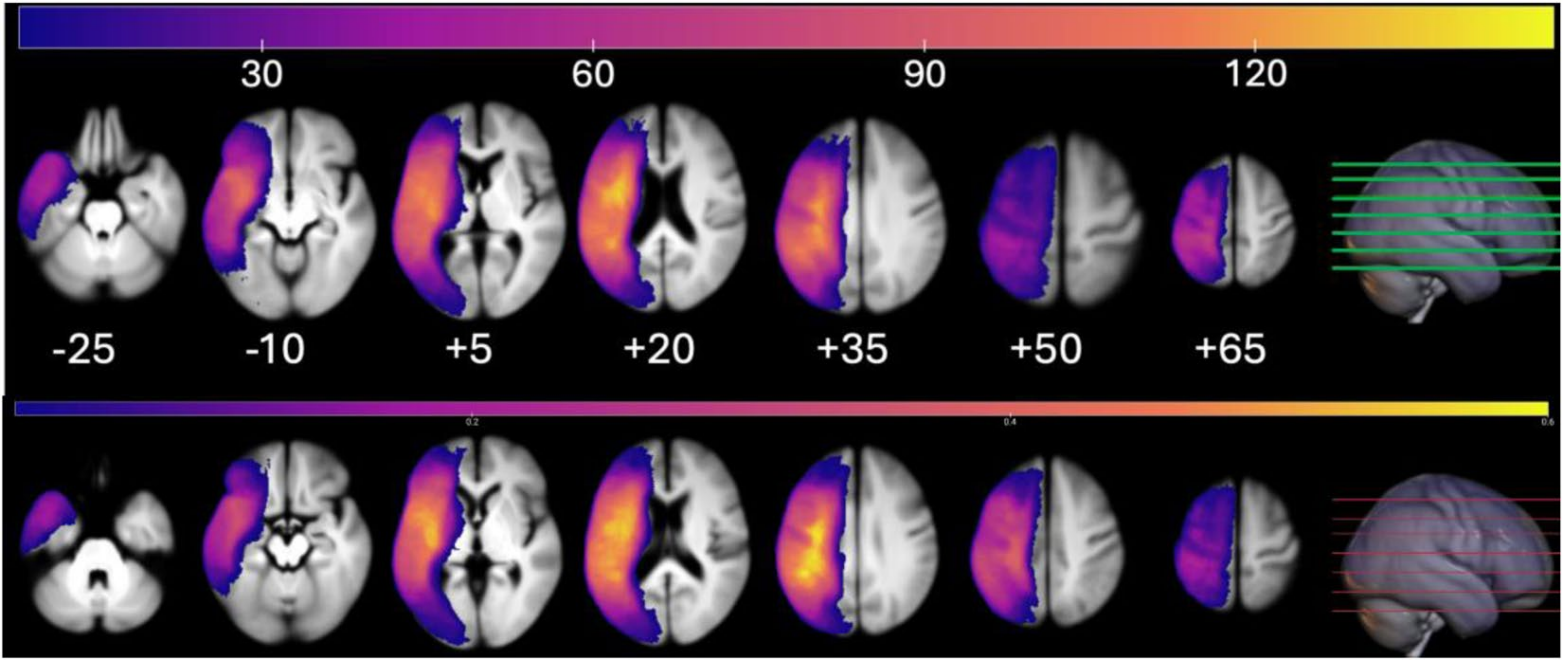
Lesion incidence map (in the Plasma color scheme) shown on top of the mean anatomical image (grayscale). As expected for studies of aphasia, all individuals had left-hemisphere strokes, typically confined to the middle cerebral artery territory. The colorbar shows the number of participants with a lesion to a specific voxel. Lower numbers indicate the z-axis position of axial slice in standard MNI152 space.

Our scripts also concatenate data across individuals and create mean lesion-incidence and anatomical images (Fig. 2). We provide a script that quantifies the amount of injury in an atlas for each individual, providing a measure of lesion load for subsequent analyses. While this script can use any atlas that is aligned to the images, we provide the Arterial Atlas^57^. In addition, this script allows brain regions that are virtually never injured to be excluded for future analyses, allowing the user to set a threshold for sufficient lesion affection^58^ providing a simple form of feature selection. We also provide a script that concatenates behavioral (performance on the WAB) and relevant demographic variables^57,59^ (e.g., participant age) with the lesion-load measures.

**Fig. 3:**
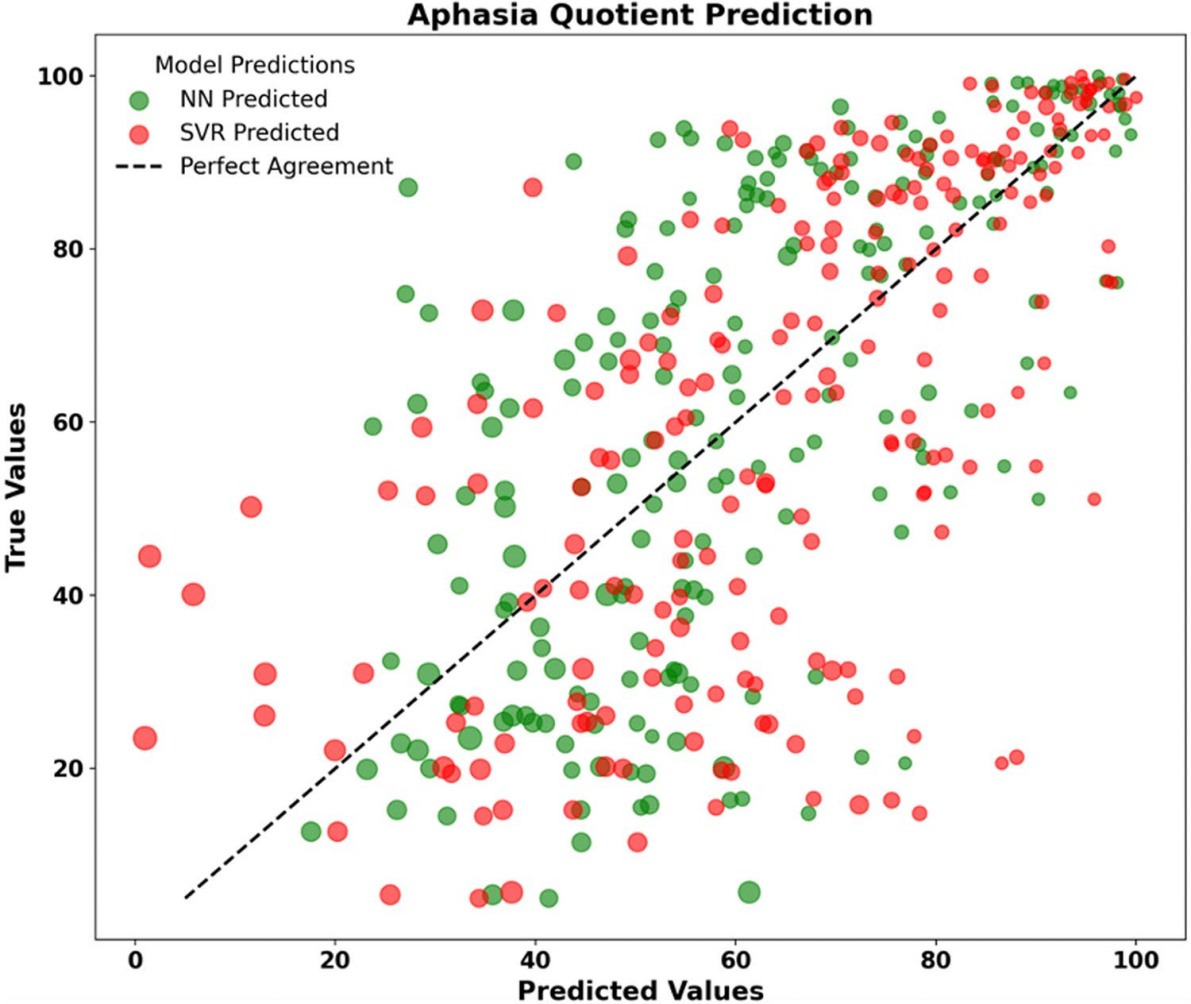
Prediction of the response variable WAB-AQ using total lesion size, proportional injury to the vascular territory atlas described by Faria and colleagues^26^, and age at stroke as features. Performance of both a sequential neural network (NN) architecture and traditional support vector regression (SVR) approach are illustrated. The diagonal dashed line represents perfect agreement between predicted and actual values. Data point size is a function of z-score transformed lesion size, proportional injury to the vascular territory atlas, and age as features. Performance of both a sequential neural network architecture and traditional support vector machine approach are illustrated.

We also provide a script that uses machine learning to predict the impairment on the WAB leveraging features from brain imaging data as well as relevant demographic features. The analysis utilizes a TensorFlow-based implementation in Python, employing a sequential neural network architecture. The model consists of three layers: an initial dense layer with 64 nodes and a rectified linear unit (ReLU) activation function, followed by a second dense layer with 32 nodes and ReLU activation, and finally, an output layer with a single node. We also provide an identical analysis using support vector machines, which can sometimes be more robust for relatively small datasets. In our sample, both Neural Networks (Correlation R = 0.636402956, p < 0.000001) and support vector regression (Correlation R = 0.6385465365, p = 0.000001) prove to be able to robustly predict impairment (Fig. 3). Our example demonstrates that for the initial tranche of 228 individuals, the machine learning performance is similar when provided with just a single measure of lesion volume versus the proportion of injury to each brain region. This underscores the potency of using lesion volume to predict impairment^58,60^, but may also reflect the limitations of our simple model and the currently still modest dataset size, for machine learning purposes.

This educational script was intentionally kept as simple as possible to illustrate the data, and relies on open source Python scripts and packages. However, a major aspiration for the sharing of this dataset is that others will exploit it to improve each stage of processing, including: lesion mapping, spatial normalization, brain extraction, and impairment prediction.

## Data Records

The Aphasia Recovery Cohort dataset uses the BIDS format to organize and name files and folders and is accessible on the OpenNeuro web site (10.18112/openneuro.ds004884.v1.0.1)^61^. The data has been anonymized and defaced to meet the Safe Harbor guidelines. The data will be released in two batches, which allows competitions between teams to estimate performance on hidden data. The initial release provides data from 230 unique individuals scanned during 902 sessions. Sessions were acquired on average 1638 days post stroke (range: 56..9685, standard deviation = 1646). Modalities include T1-weighted (229 individuals, 441 series), T2-weighted (229, 447), FLAIR (138, 235), tractography-capable diffusion (217, 2089), functional (193, 894), and resting-state (192, 508) modalites.

Among these, 230 individuals completed scanning from the first session, with these images acquired on average 1138 days post-stroke (standard deviation = 1327 days), 141 underwent scanning in a second session on average 1302 days post-stroke (standard deviation = 1358), and 103 individuals participated in a third scanning session on average 1308 days post-stroke (standard deviation = 1250). The average number of days between participants’ first and second sessions averaged 207 days (standard deviation = 418 days).

## Technical Validation

The ARC encompasses a rich dataset with numerous modalities. The raw DICOM data was converted to BIDS using dcm2niix^62^. All anatomical scans have facial features removed using the spm_deface algorithm (included in the Statistical Parametric Mapping [SPM] package^63^). Each of these scans was inspected visually with volume rendering to ensure the successful removal of identifying features. An expert drew a lesion map on the T2w scan from the first visit. The median lesion volume of the cohort was determined to be 69.2 cubic centimeters. We used the BIDS validator (v1.13.0; 10.5281/zenodo.3688707) to ensure that the dataset matches the BIDS specification.

## Usage Notes

The ARC is publicly shared on OpenNeuro using the community developed BIDS structure to enable usage with any BIDS-compatible pipeline. We hope that this will encourage the development, validation, and education for novel tools that are capable of handling clinical data. The Technical Validation section describes a simple set of analyses using current best practices. The Matlab and Python scripts for reproducing these results are available from Github (https://github.com/neurolabusc/AphasiaRecoveryCohortDemo). By design, these scripts focus on simplicity for clarity and training. These scripts provide a basic validation benchmark so others can evaluate the performance of more sophisticated solutions.

### Limitations

The ARC was developed retrospectively, aggregating across a number of studies. This feature introduces additional variability that ultimately limits the statistical power.

First, all participants volunteered for aphasia therapy. While these design choices maximized the statistical power of the original studies, this does mean that the sample is specific to individuals with aphasia, and re-use of these data may be impacted by this focus. Individuals that do not have aphasia following a left-hemisphere infarct may help researchers identify brain areas that are not critical to developing chronic aphasia. As a corollary, this sample disproportionally includes individuals with aphasia, with the injuries biased toward the middle cerebral artery territory.

Second, the data was acquired across multiple years and thus saw progress in MRI hardware, software, and sequence design. This can increase variability in cross-sectional studies but could also introduce a more insidious impact on longitudinal studies. Likewise, the goals of different studies influenced the sequences. For example, the number of slices for the T1-weighted images was increased in brain stimulation studies to provide accurate scalp coregistration. Likewise, the diffusion-weighted sequences for some studies were focused on high spatial resolution for accurate tractography, while other sequences used lower resolutions and higher b-values to provide stable measures of integrity, such as kurtosis. While this variability might be useful to ensure robust tool development, it does impact the power of studies hoping to re-use this dataset.

Another potential limitation of the ARC is that it only provides chronic imaging and measures. The sample does include many individuals observed at multiple time points, which is sufficient to observe some variability in chronic trajectory^64^. However, the lack of acute information does limit the inference that one can draw regarding acute-to-chronic recovery. Studies that include both acute and chronic measures can provide important insight regarding prognosis, whether treatment should focus on rehabilitation or compensation, and ultimately can help counter-balance interventional trials by matching individuals across treatment arms who have similar expected trajectories (minimizing the variability in these studies).

## Data Availability

No custom code was used.
